# Empirical research on teacher competence in mathematics lesson planning: recent developments

**DOI:** 10.1007/s11858-023-01487-2

**Published:** 2023-04-27

**Authors:** Mustafa Cevikbas, Johannes König, Martin Rothland

**Affiliations:** 1 University of Hamburg, Von-Melle-Park 8, 20146 Hamburg, Germany; 2 University of Cologne, Gronewaldstr. 2a, 50931 Cologne, Germany; 3 University of Münster, Bispinghof 5/6, 48143 Münster, Germany

**Keywords:** Competence, Dispositions, Lesson planning, Lesson study, Mathematics teaching, Systematic review, Teacher education

## Abstract

Lesson planning is of central importance to the teaching of all subjects in school. However, despite its high relevance, there is still a substantial need for a comprehensive review of factors affecting lesson planning. Empirical evidence on how teachers’ competence in lesson planning can be developed, what challenges may be encountered during the lesson planning process, and successful lesson planning designs and practices should come to light on. To close this gap the current paper presents the results of a systematic review of 20 empirical research studies on teacher competence in mathematics lesson planning. For detailed insight into the most recent contributions of the reviewed studies on mathematics lesson planning, we analyzed studies conducted during the past decade, adapting the “process model of lesson planning” and the model of “competence as continuum” as a heuristic for approaching lesson planning. We present key results of the studies related to four major themes: (1) dispositions and their influence on developing and implementing lesson plans, (2) quality aspects of lesson plans and the development of lesson planning skills, (3) difficulties in lesson planning, and (4) the relationship between lesson planning skills and performance in implementation of lesson plans. The results of our literature review reveal that teachers (especially novice teachers) face difficulties in lesson planning and their overall competence (and knowledge) are not at an expert level. However, as the results of the examined studies pointed out, teachers can acquire such competence and knowledge through training within initial teacher education and professional development. Overall, teachers need support in planning mathematics lessons by delineating their lesson plan to improve their awareness of students’ thinking, possible learning trajectories, effective usage of the curriculum and teaching resources, and the potential of innovative pedagogies that incorporate new technology.

## Introduction

Lesson planning is crucial for the implementation of effective and quality-oriented teaching of all subjects in school (König et al., 2021). It is an important part of many initial teacher education programs worldwide, especially in school practical activities (Munthe & Conway, 2017). However, until now, only a few studies have evaluated pre-service teachers’ (PSTs’) and in-service teachers’ (ISTs’) lesson planning for mathematics teaching and the implementation of courses on lesson planning in initial teacher education (Morris & Hiebert, 2017). The systematic review described in this paper intends to provide an overview of the specific state of research, focusing on teaching mathematics as a core school subject, and to highlight the need for increasing empirical research on lesson planning in mathematics education in the future.

In European didactical approaches to mathematics, lesson planning traditionally plays an important role. Subject-based didactical reflections on the topics of mathematics lessons are seen as the core of mathematics didactics, which is understood as the transformation of mathematical topics to the school level through necessary simplifications (Brousseau, 2002; Kirsch, 1977; for an overview, see Blum et al., 2019; Jahnke & Hefendehl-Hebeker, 2019). These theories have been shaped by Klein’s approach, explored in “Elementary Mathematics from a Higher Standpoint”, which elucidates the connection between university mathematical knowledge and knowledge necessary for teaching and argues that all teachers should possess a sound mathematics background connected to school mathematics (Klein, 1924–1928/2016).

In accordance with these theoretical approaches, lesson planning activities should be based on deep subject-related reflections (Shulman, 1987). Within the context of lesson planning, didactical analyses are emphasized in order to provide a sound basis for the lesson plan. These analyses include analysis of the content to be taught, evaluation of students’ pre-understanding, and development of a teaching approach and measures to examine students’ learning progress (Klafki, 1995; Wittmann, 1974).

Few research studies have provided empirical results on this topic (König & Rothland, 2022, 2022; König et al., 2020, 2021). A couple of studies have pointed out that, in their lesson planning, mathematics teachers mainly focus on the selection and construction of adequate tasks and anticipation of students’ work (Wengert, 1989; Yinger, 1980). Empirical studies reported that, for mathematics teachers, content-related didactical aspects were hardly important during lesson planning. It was important to generate and use tasks that were motivating and interesting for students and that could be used to develop the intended mathematical content in an appropriate way (Jaschke, 2016). Consistent with these results, other studies reported that experienced teachers did not develop elaborate lesson plans or rely heavily on curriculum programs. Instead, they utilized teaching strategies, disciplined improvisation, information about students’ knowledge and anticipated learning outcomes, and formal and informal assessments to drive instruction (Hatch & Clark, 2021). Despite the low importance that experienced teachers place on developing formal lesson plans, an expert-novice comparison revealed that expert teachers’ performance in lesson planning featured more fluency and efficiency, more concentration on designing the learning process, and careful selection of students’ activities (Li & Zou, 2017).

There are significant cultural differences in the role and understanding of lesson planning. In East Asia, lesson planning is an integral part of the educational approach of lesson study, a topic covered by another review in this special issue. Lesson study is a comprehensive approach and needs to be distinguished from lesson planning. It comprises not only the development of joint lesson plans but also joint implementation by a group of teachers. In addition, the participating group of teachers reflect afterwards and, possibly, carries out a replication. There are slight differences between the Chinese and Japanese approaches; for an overview, see Huang and Shimizu (2016).

Lesson study is strongly shaped by the East Asian teaching culture and is connected to the practice of strong mentoring activities for early career teachers (Kaiser & König, 2019). In mathematics education, it is often combined with problem-solving approaches (Gu & Gu, 2016). Due to cultural differences, there are difficulties to introduce lesson study into Western educational systems, despite many attempts. Cultural practices, such as critical lenses, must be introduced for lesson study to be effectively implemented (e.g., Bjuland & Mosvold, 2015; Fernandez et al., 2003; Groves et al., 2016).

From a more general educational perspective, within the discourse on PSTs’ and ISTs’ professional development, it is currently an open question whether lesson planning can be conceptualized as a single competence construct, as part of teachers’ professional competence, or as the product of complex learning processes leading to a multidimensional conceptualization of teacher competence (for an overview of the discourse, see Blömeke et al., 2020; König et al., 2020; Rothland, 2022). Departing from the process model of lesson planning offered by Yinger (1980) and the “competence as continuum” theoretical approach proposed by Blömeke et al. (2015), which has been influential in the current discourse on teacher professionalism (Kaiser & König, 2019), we describe lesson planning as part of teachers’ competence. This competence consists of cognitive (e.g., professional knowledge necessary for lesson planning) and affective dispositions (e.g., teacher beliefs); situation-specific abilities and skills (including perception, interpretation, and decision-making in the context of lesson planning); and performance in teaching (including implications and evaluations of the lesson plans in practice; Blömeke et al., 2020; König et al., 2020).

## Theoretical framework and research questions

A lesson plan is analogous to a road map “which describes where the teacher hopes to go in a lesson, presumably taking the students along” (Bailey, 1996, p.18). A generic definition of lesson planning is provided by Jalongo et al. (2007), who states that lesson planning can be a recursive, dynamic, cyclical, and somewhat improvisational process. According to this approach, lesson planning commences with the design of the lesson, followed by planning and implementation, then a review of the learners’ responses, and eventually concludes by circling back to the redesigning the lesson (Jalongo et al., 2007). Lesson planning involves deciding on the content and learning outcomes, identifying teaching and learning strategies, determining the assessment strategies, and evaluating the effectiveness of the lesson (Killen, 2015). Moreover, lesson planning is a critical aspect of effective teaching, as it helps teachers organize their thoughts and materials, create a clear and concise plan for instruction, and ensure that students are engaged and learning in meaningful ways (McTighe & Wiggins, 2013).

Although lesson planning is likely treated differently in various educational systems (Li et al., 2009), it is a key element of the mathematics teaching cycle since teachers and classrooms rarely run effectively without lesson planning (Yinger, 1980). Effective planning of teaching necessitates “what to teach, how to represent it, how to question students about it and how to deal with problems or misunderstanding” (Shulman, 1986, p. 8). Lesson plans help teachers to resolve instructional problems and difficulties (Richard, 1998) and allows for the creation of meaningful and purposeful learning experiences for students (Panasuk et al., 2002).

Well-thought-out lesson plans can serve as a leverage for improvement of the instructional quality (Stein et al., 1996) and provide a strong foundation for classroom implementation (Li et al., 2009). When creating high-quality lesson plans, effective teachers, as a decision maker and a problem solver, should consider the sophistication and abstraction of mathematical tasks and activities that are suitable for the students’ developmental stages and plan a trajectory for students’ mathematics learning (Clements & Sarama, 2004; Simon & Tzur, 2004). In some cases, however, actual teaching may take alternative paths or result in a surprising turn of events, even if specific interactional trajectories are anticipated in lesson plans (Lee & Takahashi, 2011). Therefore, teachers should allow themselves flexibility in designing and applying lessons considering the dynamic nature of classroom teaching in reality (Farrell, 2002).

**Fig. 1:**
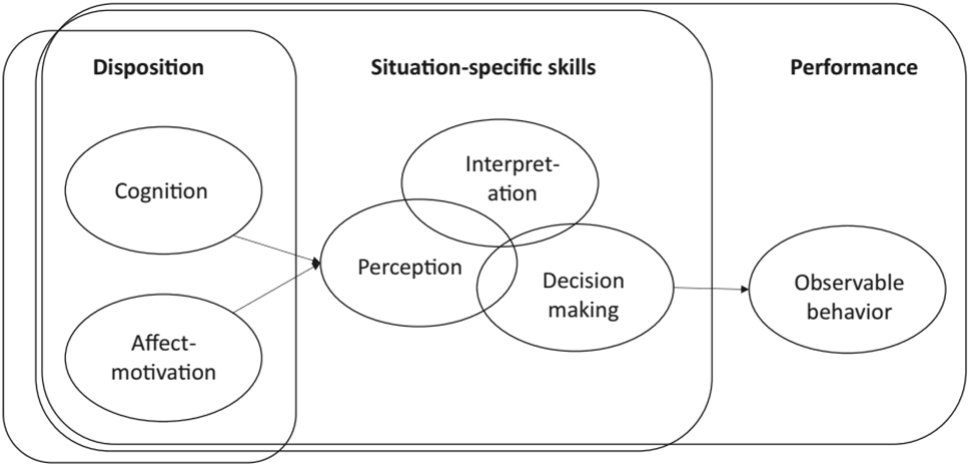
The model of competence as continuum (Blömeke et al., 2015)

**Fig. 2:**
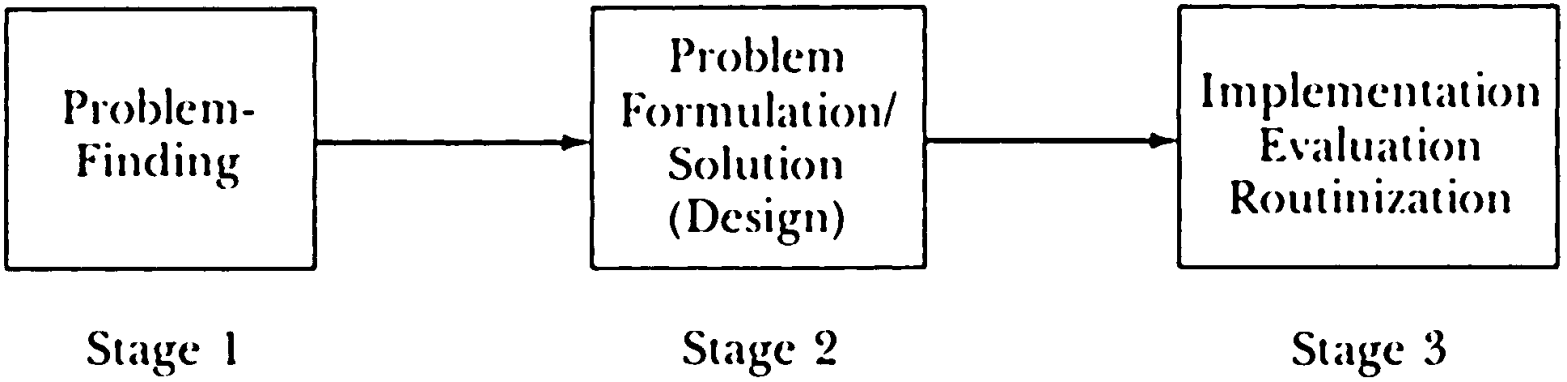
Stages of planning process (Yinger, 1980, p.114)

To provide an overview of state-of-the-art empirical knowledge on lesson planning in mathematics education and highlight the need for further research, which would be able to close research gaps, we carried out a systematic literature review. For detailed insight into the contributions of the reviewed studies on lesson planning in mathematics education, we analyzed the studies, adapting the model of “competence as continuum” (Blömeke et al., 2015, see Fig. 1) as a heuristic and Yinger’s (1980, see Fig. 2) process model of planning.

It is widely accepted that teacher competence pertains to the capability of teachers to perform their job effectively and efficiently, whereas competency is a blend of knowledge, skills, characteristics, self-perceptions, motives, values, and personal traits that empower and enable teachers to exhibit professional and effective conduct in circumstances related to teaching and learning (Blömeke et al., 2015; Metsäpelto et al., 2021). According to Blömeke et al.’s (2015) model, competence for lesson planning can be conceptualized based on mathematics teachers’ dispositions, meaning their professional knowledge (as an element of cognition) about the mathematical, mathematics pedagogical, and pedagogical bases of the subject to be taught (Klein, 2016). Affective-motivational aspects of mathematics and the structure of learning processes come into play as well (Döhrmann et al., 2012), for example, beliefs can be viewed as an affective disposition toward action or as lenses that one uses to understand certain aspects of the world (Philipp, 2007). This part of the model (dispositions, especially professional knowledge) shares similarities with the model developed by Ball et al. (2008), differences being limited to the construct conceptualization, and operationalization within these dispositional elements (Blömeke et al., 2020, p. 331).

Furthermore, teachers’ lesson planning competence in mathematics comprises situation-specific skills, which include perception, interpretation, and decision-making in the model developed by Blömeke et al. (2015). Referring to our own work (König et al., 2021) and with regards to the discourse on mathematics teacher noticing (Yang et al., 2021), we describe these three situation-specific skills as phases or sub-competences with regard to the following processes: (1) The first phase consists of, among other things, an adequate perception of current mathematical concepts, knowledge and learning needs. (2) It is followed by interpretation of the knowledge base and requirements for further development. (3) Finally, teachers must make decisions concerning an adequate lesson plan to enhance students’ learning of mathematical concepts.

Mastery of the cognitive demands of lesson planning (König et al., 2021) is characterized by (re)designing lesson plans as well as evaluation and implementation of lesson plans must be—following Blömeke et al.’s (2015) model—distinguished from actual performance in the classroom (e.g., observed teacher behavior). Performance as a broader term can be used to define the components of teacher competence as including both the ability to effectively produce and evaluate plans as well as the ability to enact those plans successfully in the classroom.

In line with Blömeke et al.’s (2015) continuum model, Yinger (1980) proposed a “process model of lesson planning” in which planning occurs in three main stages (see Fig. 2):

1. *Problem-finding:* The overall planning task is converted into a particular planning problem at this stage. Further planning and elaboration are needed when a potential instructional idea is discovered.
2. *Problem formulation/solution (design):* The initial problem-conception defined in the previous stage should be elaborated in the formulation/solution stage where the most planning time and energy are invested. Throughout this design cycle, the initial concept is continuously developed and tested in the mind until a reasonable solution is found.
3. *Implementation, evaluation, and routinization:* This stage requires implementing and evaluating the plan in the classroom. This stage informs teachers as to whether the planning activity is feasible and may lead to further modification or perhaps even rejection of the planning activity. If the activity is successful, it might gradually become routine. Results from this stage add to the knowledge and experience background, which in turn play a critical role in subsequent planning.

Yinger (1980) identifies this ongoing process characterization of the lesson planning process from conception to execution. According to this approach, each planning activity is influenced by what has come before and what may come after (Farrell, 2002). From this departure point, we examined empirical results of the studies regarding dispositions, situation-specific skills, as well as evaluation and implementation of the lesson planning holistically.

Building upon Blömeke et al.’s (2015) and Yinger’s (1980) models, we conducted a meticulous review of the literature to offer a more nuanced and comprehensive understanding of teacher competence in mathematics lesson planning. As a result, we address the following research question: “Which key empirical results have been reported by the reviewed studies on lesson planning competence in mathematics education?”.

As part of this investigation, we delve into the following sub-inquiries:

1. What dispositions do PSTs/ISTs have and how do these dispositions influence teachers’ skills/performance in developing and implementing mathematics lesson plans?
2. Which aspects of the quality of PSTs’ and ISTs’ lesson planning strategies for fostering mathematics lesson planning are examined?
3. Which aspects of PSTs’ and ISTs’ difficulties in lesson planning for mathematics are described?
4. How are lesson planning skills related to mathematics teachers’ performance in implementation of lesson plans?

## Methodology of the study

### Literature search and article selection process

Our systematic review of lesson planning in mathematics education followed the most recent Preferred Reporting Items for Systematic Reviews and Meta-Analysis (PRISMA) guidelines to enable the transfer of search strategies and relevant results to further studies or to other disciplines (Page et al., 2021). The last search was conducted in October 2022 to identify potentially relevant literature. The search request “teach* AND plan* AND lesson” was chosen for the titles of publications. Using a truncation (*), we assured that the search would account for other endings, such as “teaching,” “teacher,” “plans,” and “planning.” The search was carried out using the Web of Science (WoS) database. To explore the most recent developments in the field of mathematics education, we focused journal articles published in the past decade with a peer review to assure quality. Our electronic database searching yielded 272 studies. The references were exported to EndNote X9, and then evaluated using exclusion criteria (EC) and inclusion criteria (IC) concerning the language (English), document type (journal articles), publication year (2013–2022), domain (mathematics education), and focal points (planning mathematics lessons and empirical research about this topic).

**Fig. 3:**
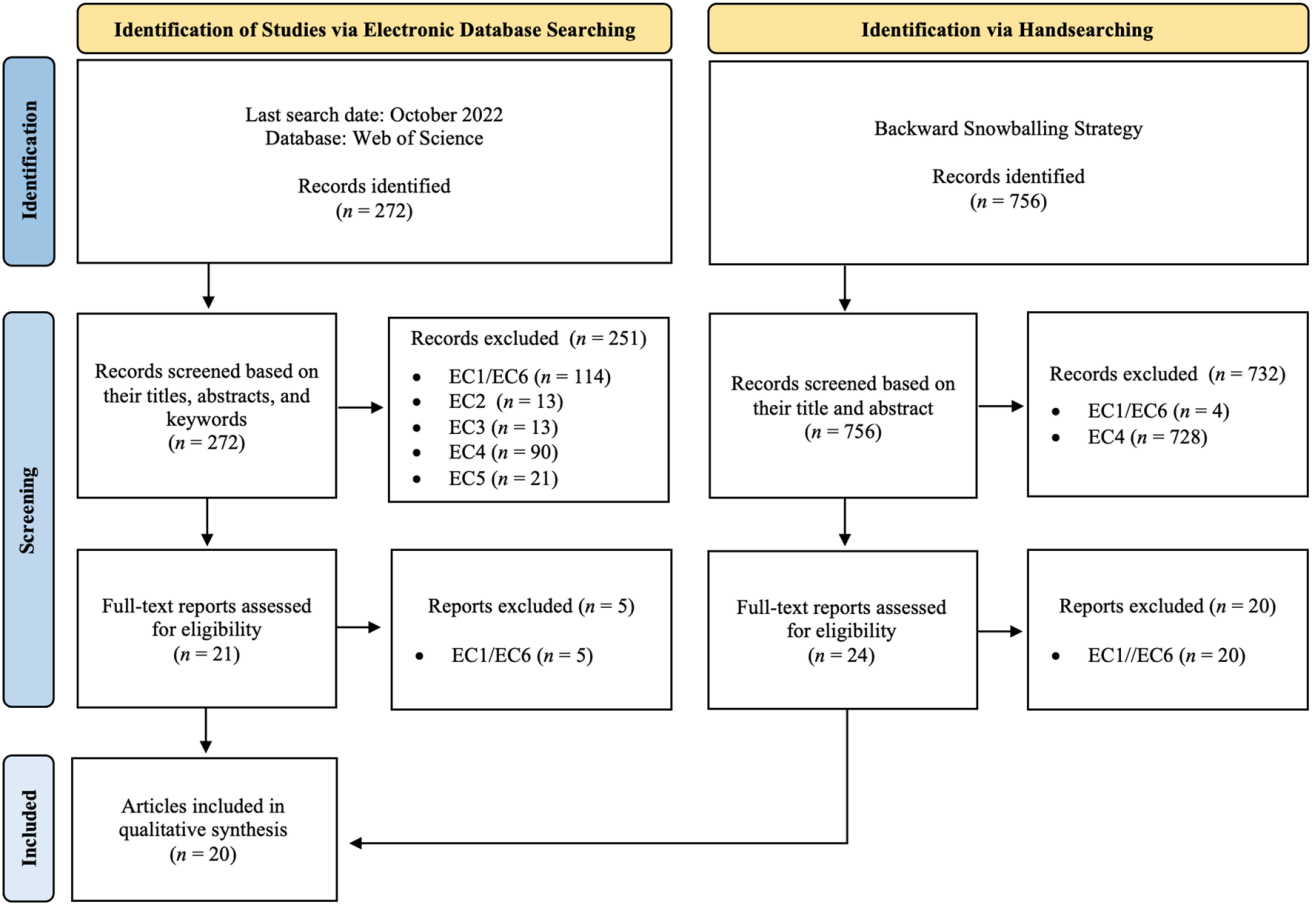
Flow diagram of the article selection process

First, we carefully screened titles and abstracts and reduced our selection to articles reporting empirical studies focused on lesson planning in mathematics education. We included studies that built their empirical investigations on designing, redesigning, implementing, or analyzing lesson plan documents/artifacts and discovering (IC1) dispositions, (IC2) quality aspects of lesson plans and strategies for fostering lesson planning, (IC3) difficulties in lesson planning, or (IC4) relationship between lesson planning and performance in teaching. During this step, we excluded articles that (EC1) had a different topic or thematic focus than PSTs’ and ISTs’ lesson planning, (EC2) were not empirical (e.g., normative guidelines for lesson planning), (EC3) were not written in English, and (EC4) were not published in the last decade, (EC5) were not indexed in educational WoS categories (i.e., educational research, education scientific disciplines, and psychology educational), and (EC6) focused on planning for lessons different from mathematics. To strengthen our repository, we carried out handsearching, using “backward snowballing” strategy, and screened 756 records that are references of the previously included studies based on our manuscript selection criteria (IC1-IC4 and EC1-EC4). Applying these criteria led to a final selection of 20 publications, which we evaluated for eligibility (see Fig. 3).

### Data analysis and reliability

The analysis included 20 articles, which are listed in Table 1 and marked with asterisks in the reference list. We first screened all articles and then encoded them based on content analysis (Miles & Huberman, 1994) with the help of a coding manual that was constructed around our research questions. We tailored the coding manual to address the specific subject matter of lesson planning, drawing upon our previous studies (Cevikbas & Kaiser, 2023; Cevikbas et al., 2022, 2023). The manual encompasses several primary categories, such as study characteristics (i.e., publication details, including publication years, authors’ affiliations, publication sources, and theoretical frameworks), research methodologies (e.g., research designs, samples, sample sizes, and data collection methods), research focus (e.g., designing, redesigning, implementing, and evaluating lesson plans), and the scope of results (including dispositions, lesson planning competence, performance in lesson planning, and the relationship between dimensions of lesson planning competence). These categories were optimized for the context of lesson planning, thus facilitating comprehensive coding of relevant study features.

**Table 1:** Included studies and the study numbers referring the related studies

Study number	Reference
1	Abadi and Ekawati (2018)
2	Amador and Lamberg (2013)
3	Backfisch et al. (2020)
4	Bieda et al. (2020)
5	Ding and Carlson (2013)
6	Earnest and Amador (2019)
7	Gonzalez et al. (2020)
8	Ozyildirim-Gumus (2022)
9	Hernandez-Rodriguez et al. (2021)
10	Lim et al. (2018)
11	Morris and Hiebert (2017)
12	Tataroglu-Tasdan et al. (2022)
13	Taylan (2018)
14	Turnuklu (2014)
15	Ulusoy and Incikabi (2021)
16	Yazgan-Sag and Emre-Akdogan (2017)
17	Akyuz et al. (2013)
18	Bremholm and Skott (2019)
19	Dunekacke et al. (2015)
20	Bauml (2014)

After completing the initial coding procedure, all reviewed articles were cross-checked by an external coder. The coder analyzed 20% (*n* = 4) of the reviewed articles based on the provided coding manual with the flexibility of offering new codes or categories. Finally, the intercoder reliability rate was found to be 92%. Although the measured reliability rate proved that the coding was reliable enough (Creswell, 2013), the discrepancies were discussed by the coders. The coding discrepancies primarily centered on the conceptualization and operationalization of dispositions. While initially, “knowledge” and “beliefs” were treated as dispositions, additional codes were identified within this category during the coding process. The coders ultimately reached an agreement on the definition of disposition posited by Taylor and Wascsko (2000), as presented in Sect. 4.1, and subsequently, a full consensus was achieved among the coders. Overall, the coding manual, coding example, as well as general study characteristics and methodologies can be found in the supplementary file in the appendix.

## Results of the study

**Fig. 4:**
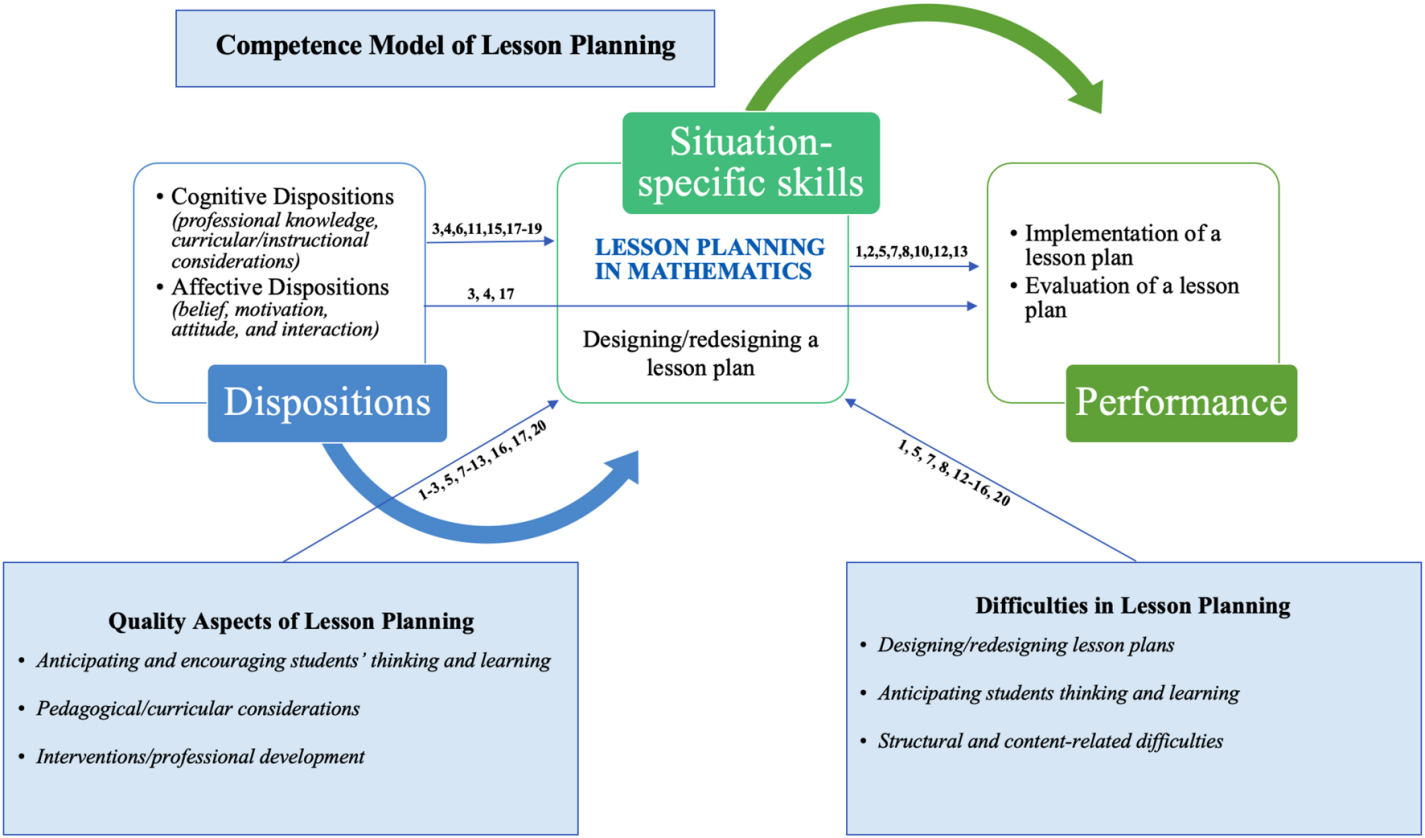
Summary of the key results integrated into the competence model of lesson planning adapted from Blömeke et al. (2015) and Yinger (1980), see Table 1 for the study numbers 1–20

In the following section, we present the key results of the reviewed studies (*n* = 20) on planning mathematics lessons in the context of the adapted competence model of lesson planning (see Fig. 4) and address the research questions. The analysis revealed that the reviewed studies contribute to lesson planning discourse in the field of mathematics education in various ways.

We present the key results of the study, which correspond to four major themes: (1) dispositions and the influence of dispositions on lesson planning and teaching performance, (2) quality aspects of lesson plans and the development of lesson planning skills, (3) difficulties in lesson planning, and (4) the relationship between situation-specific skills and performance.

In our model (see Fig. 4), we included factors that influence situation specific skills or mediators of mathematics teachers’ lesson planning competence, which include dispositions enriched by curricular/instructional considerations, motivation, affective attitude and interaction. As lesson planning competence can be measured and enhanced by aspects related to lesson planning quality, they were integrated into our model. Moreover, given that challenges in lesson planning can influence lesson planning competence in mathematics, we also considered these factors in the model. We reveal that the model we proposed is a product of the systematic literature review and it is customized for lesson planning competence beyond pre-existing models in the literature. Our aim is to offer a more nuanced and comprehensive understanding of teacher competence in mathematics lesson planning by using empirical evidence from the literature and synthesizing and building upon previous models proposed in the literature (Blömeke et al., 2015; Yinger, 1980).

### Dispositions and influence of dispositions on lesson planning and teaching performance

In Blömeke et al.’s (2015) model, dispositions refer to the potential that teachers have and bring to the instructional environment (Blömeke et al., 2020). Disposition has been defined as the personal qualities, characteristics, or tendencies that are possessed by individuals such as attitude, belief, value, interest, persistent, appreciation, enthusiasm, knowledge, understanding, interaction, collaboration, and modes of adjustment (Taylor & Wasicsko, 2000).

Our systematic review revealed that more than one third of the studies’ results (35%, *n* = 7) concerned dispositions, which have to be distinguished from lesson planning skills. We divided the reported dispositions into two major groups: (1) psychological/affective factors such as beliefs and motivational attitudes and interactions and (2) cognitive factors such as professional knowledge, understanding, and curricular/instructional considerations (e.g., classroom management and curricular work). The results of our study reveal that not only PSTs’ and ISTs’ cognitive dispositions (e.g., Akyuz et al., 2013; Backfisch et al., 2020; Bieda et al., 2020; Earnest & Amador, 2019; Morris & Hiebert, 2017), but also their affective dispositions (e.g., Akyuz et al., 2013; Backfisch et al., 2020; Bieda et al., 2020; Bremholm & Skott, 2019; Ulusoy & Incikabi, 2021) influence the situation-specific lesson planning skills and instructional quality in mathematics, particularly their skills in designing and redesigning lesson plans to account for students’ mathematical thinking, needs, and difficulties as well as important curricular issues.

Studies reviewed suggest that teachers’ dispositions, including beliefs, knowledge, motivation, affective attitudes, interactions, curricular/instructional considerations, and goals, are influential factors in the development of their lesson planning skills and practices. Teachers’ motivational conditions and their affective attitudes accounted for effective integration of technology into mathematics lesson plans, and recommended that teacher preparation programs should consider motivational aspects to foster skills in integrating technology into lesson planning (Backfisch et al., 2020). Teacher dispositions influenced lesson planning skills and shaped the structure of the plans, including preparing hypothetical learning trajectories, anticipating classroom situations and students’ reasoning, considering student engagement, and creating instructional sequences (Akyuz et al., 2013). Teachers with a higher level of content knowledge were more adept at perceiving different learning situations, planning appropriate actions, and developing effective instructional strategies, which allowed them to plan better and create more meaningful lesson plans (Bieda et al., 2020; Dunekacke et al., 2015; Morris & Hiebert, 2017). PSTs’ beliefs affected their lesson planning, leading them to heavily rely on textbooks when developing their plans (Bieda et al., 2020; Bremholm & Skott, 2019; Ulusoy & Incikabi, 2021). For example, according to the studies, PSTs believed that the textbook materials were a guarantee of well-founded instruction. Some considered that they had limited and insufficient time for creating their own original plans, which concluded approaching lesson planning superficially. Furthermore, the curriculum and interactions with school colleagues were found as influential factors on teachers’ interpretation and decision making skills in lesson planning and their instructional practices as well (Bieda et al., 2020; Earnest & Amador, 2019). However, PSTs referred to curricular elements (e.g., curricular work, classroom management, and preparational courses in teacher education) to different extents depending on their individual dispositions and educational goals when they plan lessons (Earnest & Amador, 2019).

### Quality aspects of lesson plans and strategies for fostering lesson planning

The majority of the studies (70%, *n* = 14) focused on the quality aspects of lesson planning and the development of PSTs/ISTs’ skills in designing/redesigning lesson plans. Despite their heterogeneity, a central goal of the majority of the reviewed studies was to foster lesson planning skills of PSTs/ISTs.

The results of our review revealed that PSTs/ISTs did not have mastery in designing and/or redesigning lesson plans and implementing these plans in practice, particularly at the beginning of the study interventions. However, through study interventions (e.g., teacher preparation courses, weekly content-specific planning meetings, and trainings), most participants made notable progress in their planning of mathematics lessons (e.g., Akyuz et al., 2013; Bauml, 2014; Ding & Carlson, 2013; Ozyildirim-Gumus, 2022; Hernandez-Rodrigez et al., 2021; Lim et al., 2018; Morris & Hiebert, 2017; Tataroglu-Tasdan et al., 2022; Taylan, 2018; Yazgan-Sag & Emre-Akdogan, 2017). In this way, PSTs and ISTs were able to diagnose students’ ways of learning and understanding and recognized the importance of their own content knowledge for productivity in the planning process.

A few studies reported that expert teachers performed better than novice teachers in designing and reflecting lesson plans in teaching mathematics (e.g., Amador & Lamberg, 2013; Backfisch et al., 2020; Bauml, 2014). Amador and Lamberg (2013) and Backfisch et al. (2020) revealed that especially senior ISTs could build a joint understanding of how to enhance students’ understanding, which led to lesson plans with higher instructional quality.

A few studies focused on the features of lesson plans designed by PSTs/ISTs. Studies found mixed results about the characteristics of the lesson plans. On the one hand, there was a tendency to plan lessons according to teacher-centered approach based on textbooks (e.g., Gonzalez et al., 2020; Ozyildirim-Gumus, 2022). These plans did not consider to make connection between the content, students’ previous learning, and real life situations and there was a lack of using rich instructional materials (e.g., technological tools) and deficiencies in providing appropriate feedback and formative assessment. The procedural view on mathematics was accompanied by a direct teaching approach; in other words, explanations and examples were presented before asking students to solve mathematical tasks. On the other hand, several PSTs/ISTs produced student-centered lesson plans, focusing on students’ thinking and expectations (e.g., Gonzalez et al., 2020; Ozyildirim-Gumus, 2022). In these plans, they made connections between mathematical concepts and daily life and offered to use concrete materials in teaching.

While studies have a consensus on the importance of the quality of lesson plans, they identified various strategies to produce high-quality plans, which is promising that we have many possibilities to improve the quality of lesson plans. These strategies can be divided into three groups: (1) pedagogical/curricular considerations, (2) anticipating and encouraging students’ thinking and learning, and (3) interventions/professional developments.

1. ***Pedagogical and Curricular Considerations:*** Developing a high-quality lesson plan requires incorporating effective pedagogical approaches and relevant curricular strategies. Studies have identified several considerations that can aid in this process, such as utilizing the principles of productive pedagogy within a curricular framework (Abadi & Ekawati, 2018), clarifying the lesson plan's objectives (Taylan, 2018), referencing the usefulness of mathematics and outlining assessment methods (Gonzalez et al., 2020), and utilizing a variety of rich instructional and curricular materials and resources (Lim et al., 2018). By considering these pedagogical and curricular factors, a lesson plan can be designed to effectively engage learners and meet desired learning outcomes.
2. ***Anticipating and Encouraging Students’ Thinking and Learning:*** Designing an effective lesson plan involves focusing on students’ thinking and learning situations. Studies suggest that incorporating various strategies can enhance the lesson plan quality. Anticipating classroom situations such as students’ questions, solutions, and difficulties and creating examples that meet their horizon can improve the lesson plan quality (Akyuz et al., 2013; Hernandez-Rodriguez et al., 2021; Taylan, 2018). Focusing on students’ thinking and innovative approaches in lesson planning can lead to better learning outcomes (Amador & Lamberg, 2013; Backfisch et al., 2020; Gonzalez et al., 2020; Ozyildirim-Gumus, 2022; Hernandez-Rodriguez et al., 2021; Taylan, 2018). Providing prompt questions that ask for explanation, justification, and reconsideration in lesson planning can promote critical thinking skills among students (Tataroglu-Tasdan et al., 2022). Developing hypothetical learning trajectories, designing instructional sequences, and considering the big ideas of units can enhance the overall structure and coherence of a lesson plan (Akyuz et al., 2013). By incorporating these strategies, the lesson plans can be designed to effectively address the needs of students and achieve the desired learning outcomes.
3. ***Interventions and Professional development:*** The studies we examined suggest that various interventional strategies, collaborative work, and expert support can enhance teachers’ lesson planning skills. Some of these strategies include participating in instructional interventions like trainings, seminars, workshops, and weekly planning meetings, and engaging in substantive conversations to receive feedback (Abadi & Ekawati, 2018; Bauml, 2014; Morris & Hiebert, 2017; Yazgan-Sag & Emre-Akdogan, 2017). Collaborating with knowledgeable individuals (Akyuz et al., 2013; Backfisch et al., 2020; Bauml, 2014; Ding & Carlson, 2013; Hernandez-Rodrigez et al., 2021), improving content knowledge (Abadi & Ekawati, 2018; Bauml, 2014), and utilizing digital technologies for lesson planning can also be beneficial (Backfisch et al., 2020). Additionally, receiving tailored feedback from experts and peers can be helpful (Abadi & Ekawati, 2018; Bauml, 2014; Ding & Carlson, 2013; Hernandez-Rodrigez et al., 2021), as can working to redesign pre-existing lesson plans (Lim et al., 2018).

### PSTs’ and ISTs’ difficulties in planning lessons

As mentioned earlier, our results indicated that the majority of PSTs and ISTs were not productive in designing and implementing lesson plans at least at the beginning of study interventions. For example, Ulusoy and Incikabi (2021) highlighted the difficulties experienced by PSTs in planning their own lessons. According to their results, only 30% of the participants attempted to produce their own curriculum resources for their lesson plans. Majority of the PSTs found developing a lesson plan challenging and searched on the Internet, especially in teacher portals and workbooks to find ready-to-use lesson plans and teaching activities.

Half of the studies (50%, *n* = 10) reported particular difficulties and deficiencies of PSTs/ISTs in planning mathematics lessons, which can be considered under three groups: (1) designing/redesigning lesson plans, (2) anticipating students’ thinking and learning situations, and (3) structural and content-related difficulties.

1. ***Designing/redesigning lesson plans:*** Studies found that teachers faced various challenges when it came to designing or redesigning their mathematics lesson plans. One such challenge was difficulty in creating their own lesson plans or analyzing and modifying pre-existing ones (Abadi & Ekawati, 2018; Taylan, 2018; Ulusoy & Incikabi, 2021). Another challenge was implementing a productive pedagogy to design a lesson plan, as noted by Abadi and Ekawati (2018). In addition, teachers may struggle to incorporate design elements of modern pedagogical theories and instructional principles into their lesson plans (Ding & Carlson, 2013; Gonzalez et al., 2020). Moreover, some teachers may feel unsure about developing a lesson plan or following a guided plan based on standardized curriculum, as observed by Bauml (2014).
2. ***Anticipating students thinking and learning:*** Studies showed that teachers often struggled with anticipating students’ thinking and learning patterns and integrating them into their lesson plans. We found that teachers had difficulty in noticing students’ thinking and expectations regarding mathematical topics (Tataroglu-Tasdan et al., 2022; Taylan, 2018). In addition, teachers faced challenges in acknowledging the importance of accounting for possible misconceptions among students (Turnuklu, 2014).
3. ***Structural and content-related difficulties:*** A few studies highlighted the structural and content-related difficulties that teachers faced when it came to lesson planning. According to these findings, teachers had difficulty in posing content-related problems, such as quadratic growing pattern problems, and establishing a connection between content-related topics and real-life scenarios (Ozyildirim-Gumus, 2022). Furthermore, it is observed that some PSTs misunderstood the concept of lesson planning, confusing it with the curriculum and assuming that a lesson plan was a detailed outline of the curriculum (Yazgan-Sag & Emre-Akdogan, 2017).

### Relationship between situation-specific skills and performance

As our literature review pointed out, nearly half of the studies (40%, *n* = 8) focused on PSTs’ and ISTs’ situation-specific lesson planning skills and performance, which means that the implementation of lesson planning skills in practice can be seen as a central aim of the current discourse on lesson planning. However, several studies did not provide strong evidence of a relationship between these issues. Teachers’ situation-specific skills refer to the ability to perceive and interpret what is happening in the classroom setting and then to develop instructional decisions (Blömeke et al., 2015, 2020). Especially, developing diagnostic skills as situation-specific skills are crucial to improve the quality of instructional processes (Leuders et al., 2018). They are part of decision-making for adaptive teaching (Parsons et al., 2018) and as such help teachers to master the cognitive demand of adapting to students’ learning dispositions during the planning process (König et al., 2021). Metsäpelto et al. (2021) posited that particular foundational skills of teachers may exert a direct impact on teaching practices, while others may not. Our study enriches this body of knowledge by uncovering the discernible influence of lesson planning skills on teaching practices.

The significance of foundational skills in effective teaching is further emphasized in the realm of mathematics, where Taylan (2018) underscores the importance of anticipating and diagnosing students’ thinking, misconceptions, and learning difficulties in designing and executing superior mathematics lesson plans. The reviewed studies found link between planning skills and performance, but there is also often a gap between theoretical plans and practical realities (Gonzalez et al., 2020; Ozyildirim-Gumus, 2022). The guided practices and professional support in lesson planning, combined with domain-specific knowledge, can promote teachers’ planning skills and performance (Ding & Carlson, 2013). Modifying pre-existing lesson plans is an effective strategy for improving teachers’ self-efficacy and use of planning skills in mathematics teaching (Lim et al., 2018). Experienced teachers create lesson plans in different ways and exhibit better performance than novice teachers, indicating a relationship between planning skills and instructional performance (Amador & Lamberg, 2013; Tataroglu-Tasdan et al., 2022). Developing teachers’ planning skills, including noticing skills, can contribute to the quality of mathematics teaching (Tataroglu-Tasdan et al., 2022).

## Limitations of the study

In this review, we included empirical studies written in English and published peer reviewed journals in the last decade, which probably lead to a biased representation of relevant research. Although we conducted handsearching, our electronic literature search was limited to WoS database, namely the databases other than WoS may also yield interesting studies. Among the authors of selected studies, American (45%) and European researchers (45%) predominated. Researchers from other parts of the world are underrepresented, Asia (6%) and South America (4%). This distribution of researchers may be related to the widespread use of the chosen search sequence (lesson planning) in American and European contexts and the preference of using “lesson study” in Eastern cultures. This result, on the one hand, makes transferring our results difficult to the settings outside the US and Europe. On the other hand, presented results are also important for the Eastern educational systems as the “lesson planning” is one of the core elements of “lesson study” (Fujii, 2016). Concerning the theoretical framework of the study, the model of “competence as continuum” (Blömeke et al., 2015) can serve as an overarching approach that refers to the development of competence in different fields. However, its generality can be seen also a weak point and may not solely help unpack “lesson planning” as an activity/process, as it contains its own specific aspects. Considering this limitation, we developed an enriched competence model with the focus of the lesson planning (presented in Fig. 4) motivated by the empirical evidence from the literature and two compatible well-known models: “competence as continuum” (Blömeke et al., 2015) and “process model of lesson planning” (Yinger, 1980).

## Discussion and conclusions

The present review aims to contribute to the understanding of relationships between the different constituents of lesson planning, such as affective and cognitive dispositions, lesson planning skills, and performance including implementation and evaluation of the lesson plans. Furthermore, this review presents an enriched competence model specific for lesson planning by benefiting from the models of “competence as continuum” and the “process model of lesson planning”: (1) adding new dispositions (e.g., curricular/instructional considerations, motivation, and affective attitude and interaction) to well-known dispositions (e.g., knowledge and beliefs), (2) exploring strategies to foster teachers’ planning skills and uncovering their strengths and weaknesses in lesson planning, and (3) identifying teacher performances in designing, evaluating, and implementing lesson plans in practice. Future empirical studies can use, test, and develop this enriched competence model of the lesson planning.

The results suggest that teachers’ dispositions are essential for developing lesson planning skills and successful instructional approaches. It is recommended that teacher education programs should consider fostering affective and cognitive dispositions to develop situation-specific planning skills and instructional performance in mathematics (Abadi & Ekawati, 2018; Blömeke & Kaiser, 2017; Metsäpelto et al., 2021; Seidel & Stürmer, 2014). Future studies should also explore more factors and specific intervention strategies to further develop teacher competence in lesson planning.

To improve the quality of lesson plans, reviewed studies developed and tested several useful strategies, such as conducting instructional interventions, redesigning preexisting plans, clarifying the goals and assessment tools, anticipating student thinking and learning situations, creating collaborative interactions, getting expert feedback, effective usage of the curriculum and teaching resources, and innovative pedagogies incorporating technology in lesson planning. The results suggest that professional experience and exposure to lesson plan development can also affect teachers’ planning competence. To improve teachers’ confidence, knowledge, skills, and experience in planning mathematics lessons, studies (Akyuz et al., 2013; Bauml, 2014) suggest attending content-specific planning meetings and strengthening expert-novice relationships through collaborative interactions and sharing experiences, reflections, and anticipations. This highlights the importance of implementing various strategies to foster high-quality lesson planning that may enable successful instruction. The reported results may help teachers engage in curricular and instructional design processes and to develop their knowledge and skills in mathematics lesson planning as well. Further research should investigate additional methods to support teachers in developing their lesson planning skills and successful enactment of lesson plans. Future researchers can extend the list of the identified strategies to develop high-quality lesson plans by focusing on different cultural and educational contexts. Cross-cultural and intercultural studies may yield interesting empirical results on mathematics teachers’ competence in lesson planning and how to develop their planning skills.

Our study reveals that teachers face difficulties in planning lessons, and their overall competence are not at an expert level (e.g., skills and performance in lesson planning featured less fluency and efficiency, less concentration on designing the learning process, and careless selection of students’ activities; Li & Zou, 2017). Teachers, especially novice teachers, face difficulties in lesson planning, particularly in the successful integration of all aspects of Blömeke et al.’s (2015) model. Therefore, teachers need a comprehensive theoretical foundation and reflective experience in the field to become proficient in lesson planning. Courses in teacher education or professional development programs can provide an instant response to this issue. Large-scale studies can provide a big picture, helping to identify common problems in lesson planning and teachers’ overall strengths and weaknesses in planning a lesson and its applications. Future research can develop and evaluate useful strategies for how mathematics teachers can overcome these reported difficulties in lesson planning. The identified difficulties in lesson planning and shortcomings of teachers may be rooted in the lack of familiarity and experience with lesson planning (Tataroglu-Tasdan et al., 2022). Moreover, there may be a link between these results and the dispositions; for example, the lack of teacher knowledge and motivation as well as negative beliefs about the value of lesson planning may negatively affect teachers’ lesson planning processes. In addition, teachers’ professional backgrounds and their old habits might be among the reasons for the reported difficulties (Ozyildirim-Gumus, 2022). However, the results of the reviewed studies do not allow us to propose comprehensive explanations, as most of them do not provide sufficient background information of their participants. These reported difficulties in lesson planning faced by teachers need to be addressed in further empirical studies in order to identify strategies to overcome existing problems in planning mathematics lessons.

Moreover, the complex chain from situation-specific skills to performance has to be taken into account, whereby their connections cannot be taken for granted as the processes highlighted in the model by Yinger (1980) suggest: Due to “double contingency” in the social system of classroom teaching (König et al., 2021, p. 469), implying that teachers cannot always be certain of the effects of their actions in the classroom, teacher performance is not preemptively decidable – neither through their professional knowledge nor their lesson planning skills. Teacher educators should balance theory (design principles of the lesson plans) and practice (implementing lesson plans) when teaching courses on lesson planning for PSTs or providing professional development activities for ISTs (Metsäpelto et al., 2021).

In addition, our review produced limited evidence for the role of digital technologies in mathematics lesson planning. Future studies can investigate either how digital technologies can contribute to the lesson planning process (König et al., 2022) or what kind of challenges teachers have to master, taking into account the experiences of teachers in technology-supported mathematics instruction, especially during the COVID-19 pandemic (Cevikbas & Kaiser, 2023).

Overall, this systematic review study helps us to understand the complexity and dynamics of teacher competence in mathematics lesson planning, which may inspire future research studies. It may also contribute to the development of effective professional development programs and teacher training initiatives aimed at improving teachers’ lesson planning competence.
